# Transcriptome Analysis in Patients with Chronic Kidney Disease on Hemodialysis Disclosing a Key Role for CD16^+^CX3CR1^+^ Monocytes

**DOI:** 10.1371/journal.pone.0121750

**Published:** 2015-04-01

**Authors:** Eva Schepers, Erica Houthuys, Annemieke Dhondt, Grim De Meyer, Nathalie Neirynck, Pascale Bernaert, Rafael Van den Bergh, Peter Brouckaert, Raymond Vanholder, Griet Glorieux

**Affiliations:** 1 Department of Internal Medicine, Nephrology Division, Ghent University Hospital, Ghent, Belgium; 2 Unit for Medical Biotechnology, Inflammation Research Center (IRC), VIB and Laboratory for Protein Biochemistry and Biomolecular Engineering, Department of Biochemistry and Microbiology, Ghent University, Ghent, Belgium; 3 Department of Internal Medicine, Cardiology Division, Ghent University Hospital, Ghent, Belgium; 4 Renal Division, AZ Maria Middelares, Ghent, Belgium; 5 Department of Molecular and Cellular Interactions, VIB—Vrije Universiteit Brussel, Pleinlaan 2, B-1050 Brussels, Belgium; 6 Laboratory of Cellular and Molecular Immunology, Vrije Universiteit Brussel, Pleinlaan 2, B-1050 Brussels, Belgium; 7 Department of Biomedical and Molecular Biology, Ghent University, Zwijnaarde, Belgium; Morehouse School of Medicine, UNITED STATES

## Abstract

The risk for cardiovascular morbidity and mortality is increased in chronic kidney disease; in this process micro-inflammation plays an essential role. Responsible mechanisms remain to a large extent unidentified. In this pilot study transcriptome analysis of peripheral blood monocytes was used to identify in an unprejudiced manner which factors could be discriminative for cardiovascular disease in patients with chronic kidney disease on hemodialysis. Forty gender- and age-matched, non-diabetic, non-smoking subjects with CRP < 20 mg/L were recruited: 9 healthy controls, 11 patients with eGFR > 60 mL/min/1.73m^2^ and a history of cardiovascular event (CVE), 10 patients with chronic kidney disease stage 5 on hemodialysis without previous cardiovascular event (CKD5HD) and 10 with a previous cardiovascular event (CKD5HD/CVE). Monocytes were isolated and their mRNA was submitted to focused transcriptome analysis using a macroarray platform containing ca. 700 genes associated with macrophage functional capacity. The macroarray data indicated 9 genes (8 upregulated and 1 downregulated) with a significant differential expression in CKD5HD/CVE vs. CVE alone, after excluding genes differentially expressed in CKD5HD vs. control. For FCGR3A (CD16) and CX3CR1 (chemokine receptor) the upregulation vs. control and vs. CVE could be confirmed by quantitative RT-PCR for all CKD5HD patients. Furthermore, CX3CR1 relative expression on monocytes correlated with CRP. Flow cytometric analysis of purified monocytes confirmed a significant increase in the percentage of CD16 positive monocytes in all CKD5HD patients vs. control and CVE. The present study indicates the importance of a specific pro-inflammatory monocyte subpopulation, positive for CD16 and the co-expressed chemokine receptor, CX3CR1, discriminative for CKD5HD patients.

## Introduction

Chronic kidney disease (CKD) is a well known condition of chronic low-grade inflammation contributing to the accelerated progression of organ damage of which cardiovascular disease is a major component and the main cause of death [1,2]. Next to traditional risk factors, which cannot fully explain the accelerated atherogenesis, non-traditional risk factors contribute to the inflammatory and cardiovascular burden [3]. Nevertheless, causes and patho-physiological mechanisms are still insufficiently known. Leukocytes play an important role, from the early developmental phase of vascular disease on [4]. In uremia, basal activation of leukocytes [5] has been linked to microinflammation, malnutrition and atherosclerosis [6]. Uremic retention solutes and renal replacement therapy contribute to many essential alterations of leukocyte function such as in oxidative burst, chemotaxis, apoptosis and cytokine production [7–12] demonstrating that leukocytes play a key role in uremic vascular disease [13]. Among leukocyte subtypes monocytes have an important role in every stage of atherogenesis [14].

Recently, attention has been drawn to the implication of distinct monocyte subtypes in atherosclerosis, although their functional roles are not fully understood [15]. Both CKD and coronary artery disease are associated with changes in differential monocyte counts [16,17].

In the present pilot study, transcriptome analysis of the purified mRNA of peripheral blood monocytes from CKD5HD patients and healthy controls, with and without prevalent cardiovascular events, was performed to gain better insight in the particular activation state of monocytes in CKD. For our research we focused on a broad array of target mechanisms, to avoid a prejudiced and thus biased selection of potentially responsible factors. Gene expression profiling was applied using an in-home monocyte/macrophage-focused cDNA array platform [18].

## Materials and Methods

### Patients

Forty subjects were recruited: 9 healthy controls; 11 patients with cardiovascular disease defined as a proven coronary (myocardial infarction or angina), cerebrovascular (transient ischemic attack or cerebrovascular accident) or peripheral vascular event and/or a coronary or peripheral vascular intervention (both surgical and non-surgical) and an estimated glomerular filtration rate above 60 mL/min/1.73m² calculated with the CKD-EPI formula [19]; 10 patients with CKD on hemodialysis without known cardiovascular event and negative on angiography for vascular disease; and 10 CKD patients on hemodialysis with a history of cardiovascular event. In the CKD population 13 patients were treated with high-flux membranes (n = 6 in CKD5HD, n = 7 in CKD5HD/CVE) while the other 7 were on low-flux hemodialysis (n = 4 in CKD5HD, n = 3 in CKD5HD/CVE). In each group 2 patients were on nocturnal hemodialysis (8 hours, 3 times a week), while the other 8 were on regular HD (4 hours, 3 times a week). All subjects were age- and gender-matched. Patients with a C-reactive protein (CRP) plasma level of > 20 mg/L, active infection or malignancy, auto-immune disease or diabetes mellitus and current smokers or those who had stopped smoking for < 3 months before entry into the study were excluded. All subjects gave their informed, written consent. The study was approved by the local ethical committee (Ethical Committee, Ghent University Hospital, Ghent, Belgium) and performed in accordance to the ethical principles of the Declaration of Helsinki.

### Sample collection

Twenty ml of whole blood was collected in sodium heparinised Vacutainer tubes (Becton Dickinson, San Jose, CA); in hemodialysis patients sample collection occurred immediately before the start of a mid-week hemodialysis session. Leukocyte differentiation, urea, creatinine, total protein, albumin, CRP and Glomerular Filtration Rate (GFR) data were obtained using standard techniques on EDTA anticoagulated blood. Leukocytes were counted using a Coulter Particle Counter (Coulter Beckman, Brea, CA)

### Isolation of monocytes

The isolation procedure was initiated within 30 minutes after collection in heparinised tubes.

**Fig 1:**
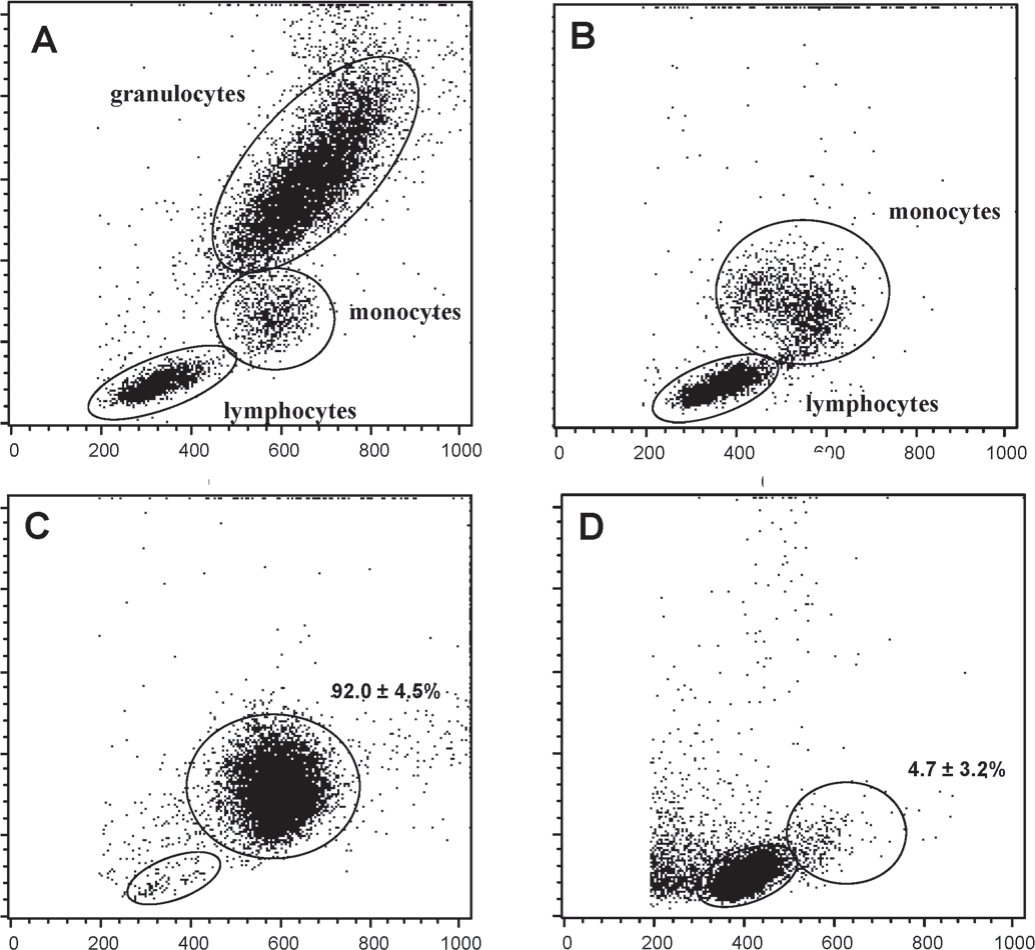
Control of the purity in the consecutive steps of the isolation procedure of monocytes. Whole blood (A); peripheral blood mononuclear cells isolated after density gradient centrifugation (B); monocytes (C) and lymphocytes (D) isolated by MACS CD14 microbeads.

Peripheral blood mononuclear cells were separated by density-gradient centrifugation using Ficoll-Paque Plus (GE healthcare Bio-Science, Uppsala, Sweden) and were further processed for separation of monocytes using Magnetic Activated Cell Sorting (MACS, Miltenyi Biotec, Auburn, USA) using the MACS Monocyte isolation kit II (see S1 Fig.) or using CD14 microbeads (Miltenyi Biotec). After every step of the isolation procedure purity was checked with flow cytometry based on forward and side scatter properties (Fig. 1). For RNA isolation, purified monocytes were lysed in TRIzol Reagent (Invitrogen Life Technologies, Carlsbad CA, USA) and stored at -80°C.

### RNA isolation, cDNA synthesis and custom cDNA arrays

Total RNA was extracted with TRIzol Reagent (Invitrogen Life Technologies, Carlsbad CA, USA) and cleaned using RNeasy Mini Kit (Qiagen, Valencia, CA). Randomly selected samples were checked for integrity on a BioAnalyser (BioRad, Hercules, CA, USA). No contamination or degradation of RNA was detected. Reverse transcription was performed on 1 μg total RNA using oligo (dT) 15 primers (Promega, Madison, USA) and Superscript II reverse transcriptase (Gibco BRL) in the presence of 33P-dCTP (Amersham GE Healthcare).

The custom cDNA array was developed as a focused and flexible tool for the analysis of gene expression patterns in monocytes/macrophages. A collection of 767 genes associated with different monocyte/macrophage activation state was compiled [18]. Subsequently, gene specific primers were designed for the genes in this collection and fragments were amplified from total cDNA pools of monocytes/macrophages under various *in vitro* and *ex vivo* conditions. The first round was performed on a cDNA pool derived from either monocytes and monocyte-derived macrophages stimulated in vitro with IFN‐γ, TNF‐α, IL4 or IL10 (to pick up abundant, ubiquitously expressed genes) or from ex vivo monocytes of HIV patients, broncho‐alveolar lavage cells of lung cancer or bronchitis patients and peritoneal dwell cells of patients with CKD, undergoing chronic ambulant peritoneal dialysis as a treatment regimen [20]. These fragments were transferred in duplicate onto 7x10 cm nylon membranes (Hybond-XL filters; Amersham GE Healthcare) and cross-linked to the membranes by UV exposure. The labeled cDNA was hybridized to the membranes for 20 h at 42°C in NorthernMax hybridization buffer (Ambion, Austin, TX, USA). Membranes were subsequently washed with SDS-containing buffer at 68°C and exposed to a phosphor screen to reveal bound radioactivity. Phosphor screens were then scanned in a phosphor-imager (BioRad, Hercules, CA). Spot recognition and quantification, background correction, and array normalization were all performed using custom-designed software based on the program ImageJ (Image Processing and Analysis in Java, Sun Microsystems, Santa Clara, CA, USA) [18].

As for most genomic assessments, significant upregulation was accepted when the fold-change between the means of two groups was ≥ 1.5, and the p-value was < 0.05, significant downregulation was accepted when fold-change was proportionally ≤ 0.67. All genes conform this definition from all pairwise comparisons were listed as supplemental data (S1 Table). However, to point out possible markers for cardiovascular disease in the hemodialysis population more stringent conditions were applied, being fold-change ≥ 2.0 (up-regulation) or ≤ 0.5 (down-regulation) and a p-value ≤ 0.01.

### Confirmation of macroarray data with quantitative RT-PCR

The RNA used for macroarray hybridisation was also used for quantitative RT-PCR. Equal amounts of RNA were reverse transcribed using iScript reverse transcriptase (Bio-Rad). cDNA was amplified using the lightCycler 480 SYBR Green I master mix (Roche, Mannheim, Germany) on a LightCycler 480 system (Roche). The average threshold cycle of triplicate reactions was used for all subsequent calculations using the ΔΔCt method [21,22]. Gene expression was normalized for the two most stable of the commonly used housekeeping genes: hypoxanthine-guanine phosphoribosyltransferase (HPRT), β-actin, glyceraldehyde-3-phosphate dehydrogenase (GAPDH), hydroxymethylbilane synthase (HMBS), beta-2 microglobulin (B2M), ubiquitin c UBC), ribosomal protein L13A (RPL13A), succinate dehydrogenase complex, subunit A (SDHA) and tyrosine 3-monooxygenase/tryptophan 5-monooxygenase activation protein, zeta polypeptide (YWHAZ). Using geNORM, UBC and YWHAZ were selected as the most stable housekeeping genes [22]. Primers used for confirmation of the macroarray data were: CD16 fwd 5’-CCTCCTGTCTAGTCGGTTTGG, CD16 rev 5’-TCGAGCACCCTGTACCATTGA; CX3CR1 fwd 5’-CCCTGAATCAGTGACAGAAAACT; CX3CR1 rev 5’-ACGGAGTAGAATATGGACAGGAA.

### Confirmation at protein level

Monocytes were stained using monoclonal antibodies against CD14 which is a monocytic differentiation antigen and part of the LPS receptor complex [phycoerythrine (PE) labelled anti-CD14 (clone TÜK4), Miltenyi Biotec] and against the Fcγ receptor, CD16 [fluorescent isothiocyanate (FITC) labelled anti-CD16 (clone VEP13), Miltenyi Biotec] and analysed by flow cytometry (FACScan, Becton Dickinson, Erembodegem, Belgium). Cell populations were analysed with the FACScan flow cytometer and the CellQuestPro software (Becton Dickinson).

Monocytes in this part of the study were subdivided into 3 distinct subpopulations, based on the expression levels of CD14 and CD16 (CD14^++^CD16^-^; CD14^++^CD16^+^; CD14^+^CD16^++^) indicated by fluorescence intensity values.

### Statistical analysis

Statistics of normalised array expression data were analysed with the GeneMaths XT software package (Applied Maths, St.-Martens-Latem, Belgium). Significance was determined by an uncorrected Mann-Whitney U test.

Statistical significance of subject characteristics, monocyte subpopulations and quantitative RT-PCR data was determined by a parametric one-way ANOVA using a parametric unpaired t-test or a non-parametric Mann-Whitney U test when applicable. The GraphPad Prism 5.01 software was used (GraphPad Software, San Diego, CA). The Benjamini & Hochberg correction was applied to calculate false discovery rate (FDR) using the R-package "stats" [23].

## Results

### Patients

The patient characteristics are shown in Table 1. Patient groups were gender- and age-matched. Blood urea nitrogen (BUN), creatinine and CRP were signifcantly higher in the groups on hemodialysis (CKD5HD and CKD5HD/CVE), while both these groups showed a significantly lower total protein and albumin level. Aspirin and statin use were higher in the groups with a history of cardiovascular disease (CVE and CKD5HD/CVE).

**Table 1:** Subject characteristics.

	**Control**	**CVE**	**CKD5HD**	**CKD5HD/CVE**
	**(n = 9)**	**(n = 11)**	**(n = 10)**	**(n = 10)**
Age (years)	63.1 ± 2.4	65.7 ± 3.4	64.8 ± 4.3	72.3 ± 2.1
Gender (M/F)	5/4	6/5	5/5	5/5
Dialysis vintage	NA	NA	61.7 (23.9–210.5)	43.2 (12.8–98.9)
(months)				
BUN (mg/dl)	15.42	18.44	63.27*** ^Ɩ^ ^°°°^	47.56*** ^Ɩ^ ^°°°^
	(11.21–21.96)	(11.21–28.50)	(42.99–96.73)	(23.83–60.28)
Creatinine (mg/dl)	0.89 ± 0.04	0.88 ± 0.05	9.53 ± 0.69*** ^Ɩ^ ^°°°^	7.95 ± 0.71*** ^Ɩ^ ^°°°^
Leukocytes(/μl)	5841 ± 285	6008 ± 490	5473 ± 538	5599 ± 398
Monocytes (%)	8.6 ± 0.6	8.9 ± 0.6	10.1 ± 0.8	8.8 ± 0.8
Lymphocytes (%)	29.4 ± 2.6	29.0 ± 2.6	29.1 ± 2.7	22.5 ± 1.7
Granulocytes (%)	62.0 ± 2.6	62.2 ± 2.3	60.1 ± 3.2	64.6 ± 3.8
Total protein (g/dl)	7.4 ± 0.1	7.5 ± 0.2	6.8 ± 0.1*** ^Ɩ^ ^°^	6.9 ± 0.2* ^Ɩ^ ^°^
Albumin (mg/dl)	4.5 ± 0.1	4.6 ± 0.1	4.1 ± 0.1*** ^Ɩ^ ^°°^	4.0 ± 0.1** ^Ɩ^ ^°°^
CRP (mg/dl)	0.1 (0.05–0.1)	0.1 (0.05–1.1)	0.7 (0.2–1.6)*** ^Ɩ^ ^°^	0.5 (0.1–1.8)*** ^Ɩ^ ^°^
GFR (ml/min/1.73m^2^; CKD-epi)	82.1 ± 4.4	82.6 ± 3.9	NA	NA
Aspirin use	2	10**	1^°°°^	5
Statin use	2	8*	4	9**

Values reported are mean ± SEM when normally distributed, otherwise median and range (in parentheses) are given.

*** *p* < 0.001

** *p* < 0.01

* *p* < 0.05 vs. control

^°°°^ *p* < 0.001

^°°^ *p* < 0.01

^°^ *p* < 0.05 vs. CVE

No significant differences were found between CKD5HD and CKD5HD/CVE.

Abbreviations: CVE: patients with eGFR > 60 mL/min/1.73m² and a history of cardiovascular event; CKD5HD: hemodialysis patients without previous cardiovascular event; CKD5HD/CVE: hemodialysis patients with a previous cardiovascular event; CRP: C-reactive protein; GFR: glomerular filtration rate; NA: not applicable

### Purification of monocytes from the peripheral blood

Monocyte isolation was performed by a positive selection of monocytes using CD14 MicroBeads. This strategy resulted in an overall92.0 ± 4.5% pure monocyte population. Fig. 1C shows a representative image for a hemodialysis patient. As illustrated in Fig. 1D (hemodialysis patient), the monocyte depleted lymphocyte fraction contained only a limited amount of monocytes, with a mean of 4.7 ± 3.2% of monocytes for all study groups. A mean total yield of 6.3 x 10^6^ ± 3.2 x 10^6^ monocytes was obtained from an initial volume of 20 mL heparinised whole blood. No significant differences in purity or yield were observed amongst the different study groups.

### Gene expression analysis of isolated monocytes in CKD5HD and CVE

**Fig 2:**
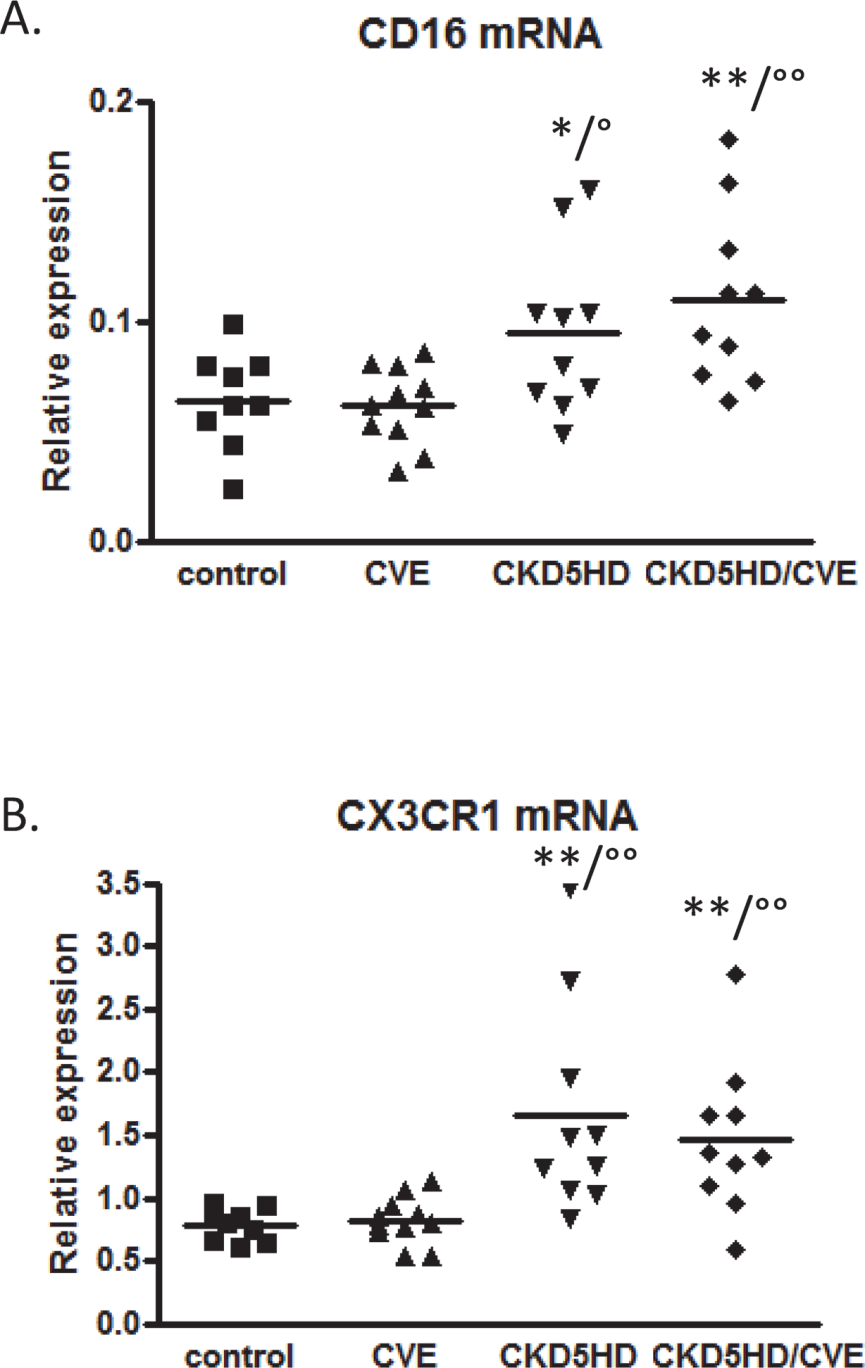
Quantitative RT-PCR data for CD16 mRNA expression (A) and CX3CR1 mRNA expression (B) in the 4 study groups; control (n = 9), CVE (n = 10), CKD5HD (n = 10), CKD5HD/CVE (n = 10). * p < 0.05, ** p < 0.01 vs. control; ° p < 0.05, °° p < 0.01 vs. CVE.

The custom cDNA array expression patterns in monocytes pointed out that gene expression related to CVE seems to be different in patients with an eGFR > 60 mL/min/1.73m^2^ compared to hemodialysis patients. Nine genes (Table 2) were revealed as candidate markers of CVE in CKD5HD patients, being significantly differentially expressed between CVE patients on hemodialysis or not (CKD5HD/CVE versus CVE), but without significant differential expression in CKD5HD without CVE versus control without CVE. These 9 genes were submitted to quantitative RT-PCR for validation of the array results. For FCGR3A (CD16) a significant upregulation could be confirmed for all CKD5HD patients as illustrated in Fig. 2A. Similar quantitative RT-PCR results were observed for CX3CR1 (Fig. 2B), which is co-expressed on CD16-positive monocytes and is involved in monocyte recruitment to and survival of monocytes in atherosclerotic plaques. The relative mRNA expression of both CD16 and CX3CR1 was significantly increased in hemodialysis patients, be it, irrespective of their cardiovascular history (CKD5HD and CKD5HD/CVE vs. Control and vs. CVE, Fig. 2A-B2). These results confirm the array results (S1 Table), where both CD16 and CX3CR1 were upregulated in all pairwise comparisons except for control vs CVE and CKD5HD vs CKD5HD/CVE. Therefore CD16 and CX3CR1 can be considered as a CKD marker in hemodialysis.

**Table 2:** List of differentially expressed genes in the comparison CKD5HD/CVE vs CVE but not in the comparison CVE vs control by the custom cDNA array in isolated monocytes with a fold-change ≥ 2.0 and a p-value ≤ 0.01.

**Symbol**	**Gene Name**	**CKD5HD/CVE vs CVE**	**p-value**
		**Fold change**	
**Upregulated**			
AK3	Adenylate b Kinase 3	2.11	0.01
CCR3	Chemokine (C-C motif) receptor3	2.74	0.001
FCGR3A	Fc fragment of IgG, low affinity	2.73	0.005
	IIIa, receptor for CD16		
IL6ST	Interleukin 6 Signal Transducer	2.49	0.002
	(gp130, oncostatin M receptor)		
PSCD1	Pleckstrin homology Sec7 and	2.05	0.006
	coiled-coil domains 1 (cytohesin 1)		
S100A8	S100 calcium binding protein A8	2.17	0.01
	(calgranulin A)		
STAT1-b	Signal Transducer and Activator of	2.30	0.003
	Transcription 1, 91 kDa		
TCEB2	Transcription Elongation factor B (SIII), polypeptide 2 (18 kDa,	2.23	0.01
	elongin B)		
**Downregulated**			
STK4	Serine/Threonine Kinase 4	0.45	0.01

### Flow cytometric evaluation of CD14 and CD16 expression on monocytes

**Fig 3:**
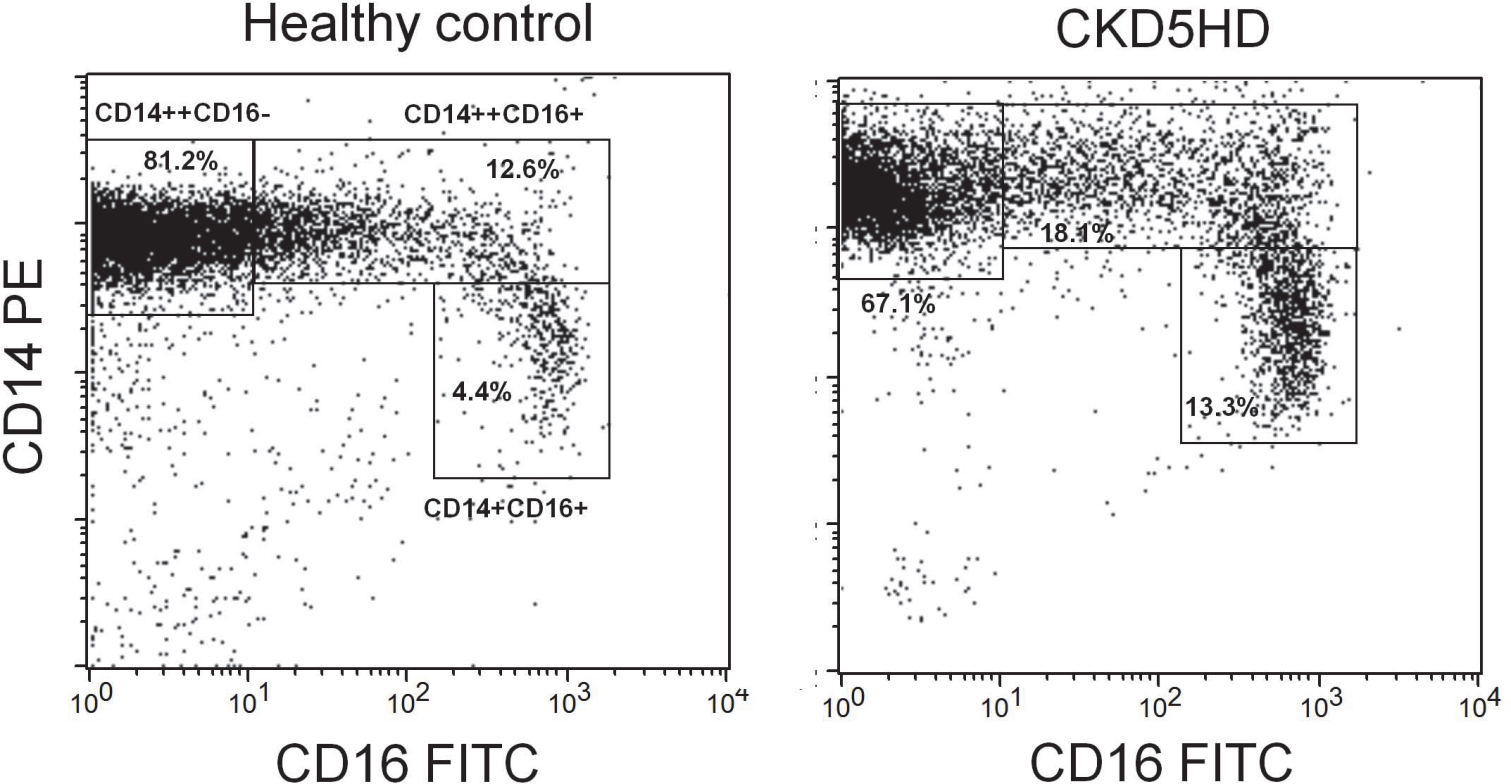
Representative sample of flow cytometric evaluation of the CD14 and CD16 expression on isolated monocytes from a healthy control (left panel) and a hemodialysis patient (right panel). According to the CD14 and CD16 fluorescence intensity three gates are drawn containing the CD14^++^CD16^-^, the CD14^++^CD16^+^ and the CD14^+^CD16^++^ monocyte subpopulations respectively. Hemodialysis patients show a decreased proportion of CD14^++^CD16^-^ and an increase of CD14^++^CD16^+^ and CD14^+^CD16^++^ monocytes.

Hemodialysis patients (CKD5HD and CKD5HD/CVE) showed a decreased proportion of CD14^++^CD16^-^ monocytes compared to patients with normal renal function (control and CVE) (Table 3 and Fig. 3). Nevertheless, the mean expression of CD14 per cell expressed as mean fluorescence intensity (MFI) was significantly higher in hemodialysis patients compared to subjects with normal kidney function (Table 3). In absolute numbers although lower, no significant difference in CD14^++^CD16^+^ cells was observed (Table 3).

**Table 3:** Percentages and absolute numbers of CD14^++^CD16^-^, CD14^++^CD16^+^ and CD14^+^CD16^++^ monocyte subsets in studied subjects.

	**Control (*n* = 9)**	**CVE (*n* = 11)**	**CKD5HD (*n* = 10)**	**CKD5HD/CVE (*n* = 10)**
% CD14^++^CD16^-^	84.4 ± 4.5	86.3 ± 3.5	62.2 ± 18.9**/°	69.7 ± 13.4**/°
CD14^++^CD16^-^/μl	415 ± 32	460 ± 53	397 ± 47	370 ± 56
MFI CD16	1.8 ± 0.5	1.3 ± 0.1	2.6 ± 1.5	3.1 ± 2.2
MFI CD 14	1393.0 ± 342.4	1365.1 ± 415.9	2196.3 ± 906.3*/°	1997.2 ± 616.3*/°
% CD14^++^CD16^+^	7.9 ± 3.4	6.8 ± 2.3	17.2 ± 10.1*/°°	15.7 ± 6.8**/°°
CD14^++^CD16^+^/μl	40 ± 6	38 ± 5	61 ± 9* ^/^ °	84 ± 14** ^/^ °°
MFI CD16	121.0 ± 24.6	121.1 ± 32.1	161.9 ± 64.7	165.2 ± 74.3
MFI CD 14	1384.2 ± 365.2	1276.1 ± 406.6	1698.2 ± 407.3°	1763.1 ± 400.3°
% CD14^+^CD16^++^	4.2 ± 2.7	5.2 ± 1.9	12.9 ± 7.2**/°°	10.4 ± 4.1**/°°
CD14^+^CD16^++^/μl	21 ± 5	28 ± 4	52 ± 9* ^/^ °	49 ± 10* ^/^ °
MFI CD16	486.8 ± 114.1	410.1 ± 80.7	418.8 ± 220.6	528.6 ± 260.7
MFI CD 14	285.1 ± 85.7	250.0 ± 83.2	313.6 ± 107.6	371.9 ± 107.7°

MFI: mean fluorescence intensity

**P<0.01

*P<0.05 vs control

°°p<0.01

°P<0.05 vs CVE

In contrast, both the proportion of CD14^++^CD16^+^ and CD14^+^CD16^++^ monocyte subsets was significantly increased in patients on hemodialysis, both in percentage as in absolute numbers, compared to the study groups with normal renal function and this in combination with a significantly increased mean expression of CD14 per cell in the CD14^++^CD16^+^ subset versus the CVE group (Table 3 and Fig. 3).

### Link to inflammation

**Fig 4:**
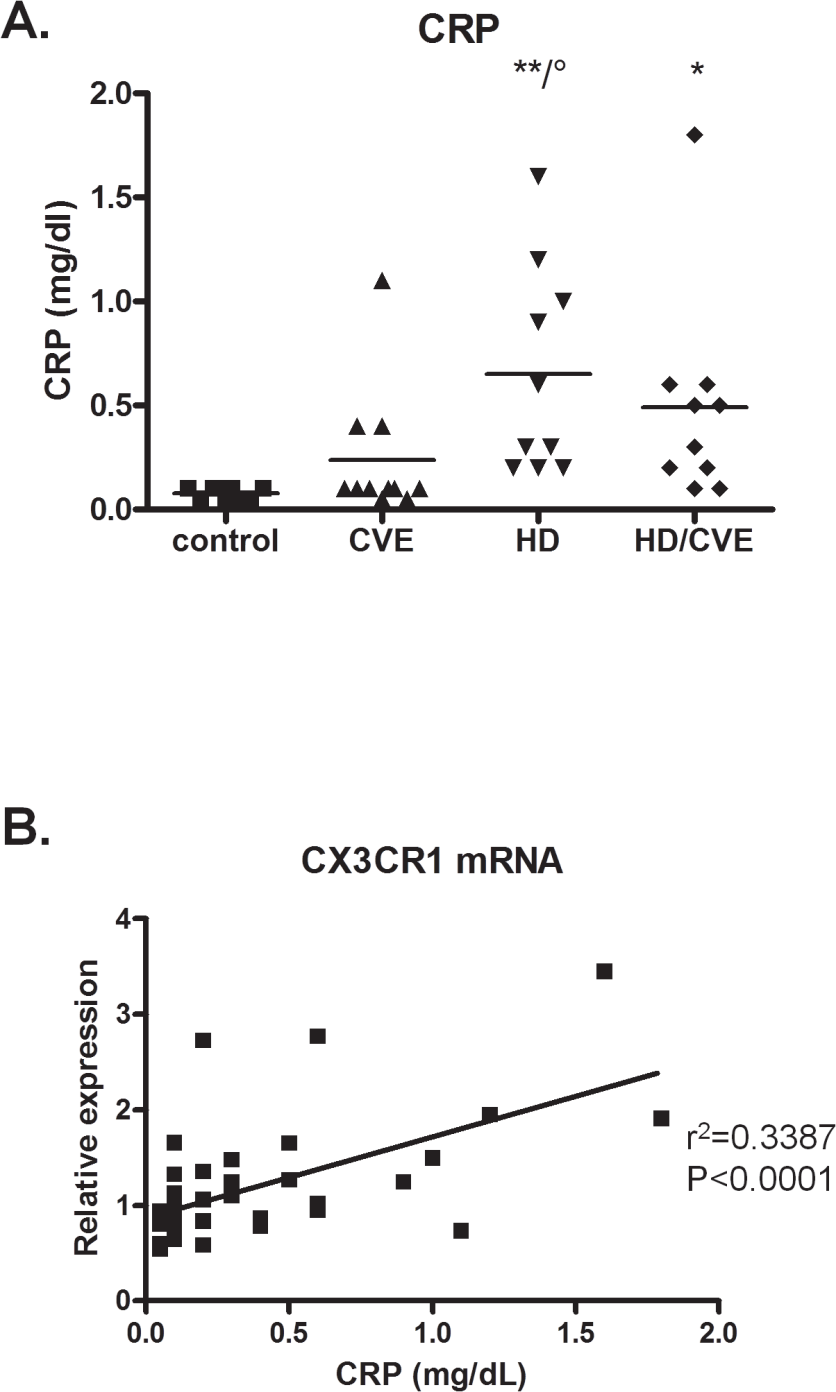
CRP levels in the 4 study groups; control (n = 9), CVE (n = 10), CKD5HD (n = 10), CKD5HD/CVE (n = 10). * p < 0.05; ** p < 0.01 vs control; ° p < 0.05 vs CVE (A). Correlations between CRP and mRNA expression (B).

Although all subjects included had a CRP level below 20 mg/L, CRP levels were significantly higher in patients on hemodialysis (Fig. 4A). In addition, the relative mRNA expression of CX3CR1 overall correlated with CRP (Fig. 4B). After adjusting this correlation for aspirin and statin intake and for type (none, coronary, cerebrovascular, peripheral or a combination) and degree (none, recovered or permanent damage) of cardiovascular damage, an adjusted R² of 0.355 was obtained where only CX3CR1 significantly (p<0.000) contributes to the CRP level.

## Discussion

In the present pilot study, transcriptome analysis of purified monocytes was used to identify factors discriminative for the basal state of micro-inflammation contributing to cardiovascular disease in patients with end stage chronic kidney disease, without applying the classical approach of selecting the parameters to study based on a prejudiced view of relevancy. To enable this, monocytes from four specific groups of subjects (chronic kidney disease patients on hemodialysis and healthy controls, both with and without a prevalent cardiovascular event) were isolated and submitted to an in-house made macroarray.

The most important findings of this gene expression analysis are that monocytes of hemodialysis patients are characterized by an increased expression of CD16 and of the chemokine receptor CX3CR1, which reflected an increased number of both CD14^++^CD16^+^ and CD14^+^CD16^++^ monocyte subpopulations in CKD patients. In addition, increased CRP levels in patients on hemodialysis correlated with CX3CR1 mRNA levels. These findings emphasize the need for studying specific leukocyte subpopulations rather than overall white blood cells when evaluating micro-inflammatory mechanisms in patients with chronic kidney disease, as the study of the global population might blur differences between small sub-populations, comprising only 1–2% of the total group of cells.

Heterogeneity of monocytes has already been described more than 2 decades ago in healthy controls [24]. Initially CD16^+^ monocytes were considered as the most pro-inflammatory subpopulation; conform with this hypothesis, their number appeared to be elevated in coronary artery disease [16], in infectious and inflammatory conditions [25] and in patients on dialysis [26]. More recently two functionally separate monocyte populations were distinguished in the CD16^+^ subset: (1) CD14^+^CD16^++^ and (2) CD14^++^CD16^+^, the latter showing more important pro-inflammatory and atherogenic features (e.g. increased inflammatory cytokine production, ROS production, migratory capacity, endothelial attachment) [27–30]. This subpopulation is increased in asthma and rheumatoid arthritis [31,32] and has been associated with subclinical atherosclerosis in renal transplant patients [33]. In patients referred for elective coronary angiography, the number of CD14^++^CD16^+^ cells independently predicts cardiovascular events [34].

In hemodialysis the CD16^+^ subtypes correlate with endothelial damage, increased phagocytosis and pro-inflammatory cytokine production [35,36]. For CKD patients on dialysis a shift towards CD14^++^CD16^+^ and CD14^+^CD16^++^ was shown [37]. Our approach, applying a blinded search, not starting from a pre-set hypothesis, for pro-inflammatory factors, thus corroborated previous findings applying predefined hypothesis-based research methods. The fact that an unblinded approach confirmed the important role of these CD16 subpopulations is to our opinion a confirmation of the likelihood that those elements play a key patho-physiologic role.

Nevertheless, for both patients not on dialysis and those treated with dialysis, CD14^++^CD16^+^ monocytes were independently associated with prevalent cardiovascular events [34,38], an association which could not be confirmed by our data. This discrepancy might be due to the fact that the present study excluded smokers, diabetics and patients with acute inflammation or infection having a CRP value above 20 mg/L. Also the significant difference in statin use in the CVE populations might be responsible for the lack of difference between CKD patients with and without cardiovascular disease, due to their anti-inflammatory properties [39]. It is of note that, due to the laborious nature of this pilot study, we included only a limited number of patients, so that the study power might have been too low to discern subtle differences. The low amount of patients might also be at the origin of loss of significance when array results were corrected for FDR (S1 Table). Moreover, fold changes were overall moderately increased. A potential cause for this observation might be due to the fact that the immune cells of CKD patients are in a chronically mild activated status [40], making it more difficult to retrieve differences in mRNA expression.

CX3CR1 is a receptor for the chemokine fractalkine (CX3CL), which promotes strong adhesion of leukocytes to activated endothelial cells, where it is primarily expressed. Ancuta et al. described a progressive increase in expression of this receptor from the CD14^++^CD16^-^ subtype, where it is almost undetectable, to CD14^++^CD16^+^ and CD14^+^CD16^++^ where the expression is highest [41]. The transendothelial migration of the latter subpopulation in response to fractalkin is in accordance with their high expression of CX3CR1. The CD16^+^ cells thus are preferentially recruited to the vessel wall and sites of inflammation via locally expressed fractalkine [27,42], which was shown to be upregulated in chronic inflammatory conditions e.g. rheumatoid arthritis [43]. Moreover, upregulation of both fractalkine and the CX3CR1 receptor were associated with coronary plaque rupture in patients with unstable angina [44]. Recently, Zaza et al demonstrated that the transcriptome profile of the total set of peripheral blood mononuclear cells including CX3CR1 levels discriminates dialysis patients from CKD stage 3–4 patients [45].

Studies comparing the transcriptome of CD16^-^ versus CD16^+^ monocyte subsets [46,47], and more recently even between the 3 monocyte populations [30,48], applying a genome-wide gene expression assay (Affymetrix, Illumina) or a serial analysis of gene expression (SuperSAGE) were by now only performed on healthy controls.

The potential drawbacks of the present pilot study are: 1) the in-house made array does not comprise the whole transcriptome, but only a focused amount of monocyte/macrophage related genes; 2) as only 10–20% of the monocytes are CD16^+^, the mRNA of the CD16^-^ subpopulation might mask potential differences in CD16^+^ cells between the study populations 3) additionally a study population of 40 subjects with 10 patients per group is rather limited to retrieve significant differences in monocyte gene expression of stable patients.

In conclusion, the present transcriptome analysis of total monocytes with a monocyte/macrophage specific array in patients on dialysis and healthy controls, with and without prevalent CV events, revealed that especially the CD16^+^ monocyte subpopulation co-expressing CX3CR1 is of importance in CKD patients on dialysis. The link to inflammation was emphasized by the correlation with the CRP levels. For the future, it is of importance to study expression profiles of human monocytes, especially in inflammatory conditions like CKD, on isolated subtypes like the most pro-inflammatory CD14^++^CD16^+^ fraction. Whether the study of isolated monocyte subpopulations with a genome-wide approach will be able to find markers that correlate with cardiovascular events in CKD populations and will gain more insight in the particular activation state of specific monocytes needs further investigation.

## Supporting Information

S1 Fig Control of the isolation procedure of monocytes isolated by magnetic activated cell sorting (MACS) monocyte isolation kit II: monocyte (A) and lymphocyte (B) fractions. For the purification of monocytes from the peripheral blood initially a negative immuno-magnetic selection strategy was applied, offering the possibility to obtain untouched monocytes. This Monocyte Isolation Kit II (Miltenyi Biotec) is based on the magnetic labeling and depletion of non-monocytes, such as T cells, NK cells, B cells, dendritic cells and basophils, resulting in pure unlabeled monocytes. When this strategy was applied on the peripheral blood mononuclear cells of a hemodialysis patient, it resulted in a monocyte population that was 93.3 ± 1.4% pure (A). However, flow cytometric analysis revealed that the remaining lymphocytic cell fraction (depleted) still contained a monocyte fraction of 8.8 ± 3.6% monocytes (B). This was due to the fact that the Monocyte Isolation Kit II contains anti-CD16 antibodies, for depletion of granulocytes and NK cells resulting in a loss of CD16 positive monocytes, a fraction of interest in the context of inflammation, since they remain in the lymphocyte fraction. Therefore this method was not used for monocyte isolation, after which we switched to a positive selection of monocytes using CD14 MicroBeads which resulted in a isolated fraction containing CD16 positive monocytes. It is of note that, due to the monocyte heterogeneity, the choice of a correct isolation procedure, resulting in only/all the cells of interest is of great importance. The aim was to study the differential expression in the total monocyte population of hemodialysis patients compared to controls and for that purpose the use of the Monocyte Isolation Kit II, resulting only in CD16^-^ monocytes, is not recommended.
(TIFF)

S1 Table Differentially expressed genes by the custom cDNA array in isolated monocytes from all pairwise comparisons with a fold change of ≥ 1.5 (up-regulation) or ≤ 0.67 (down-regulation) and a p-value of ≤ 0.05. (PDF)
