# Supplementary figures and images for: Transcriptome Analysis in Patients with Chronic Kidney Disease on Hemodialysis Disclosing a Key Role for CD16+CX3CR1+ Monocytes
Source: PLoS One. 2015 Apr 1;10(4):e0121750. doi: 10.1371/journal.pone.0121750 (PMC4382044; doi:10.1371/journal.pone.0121750)

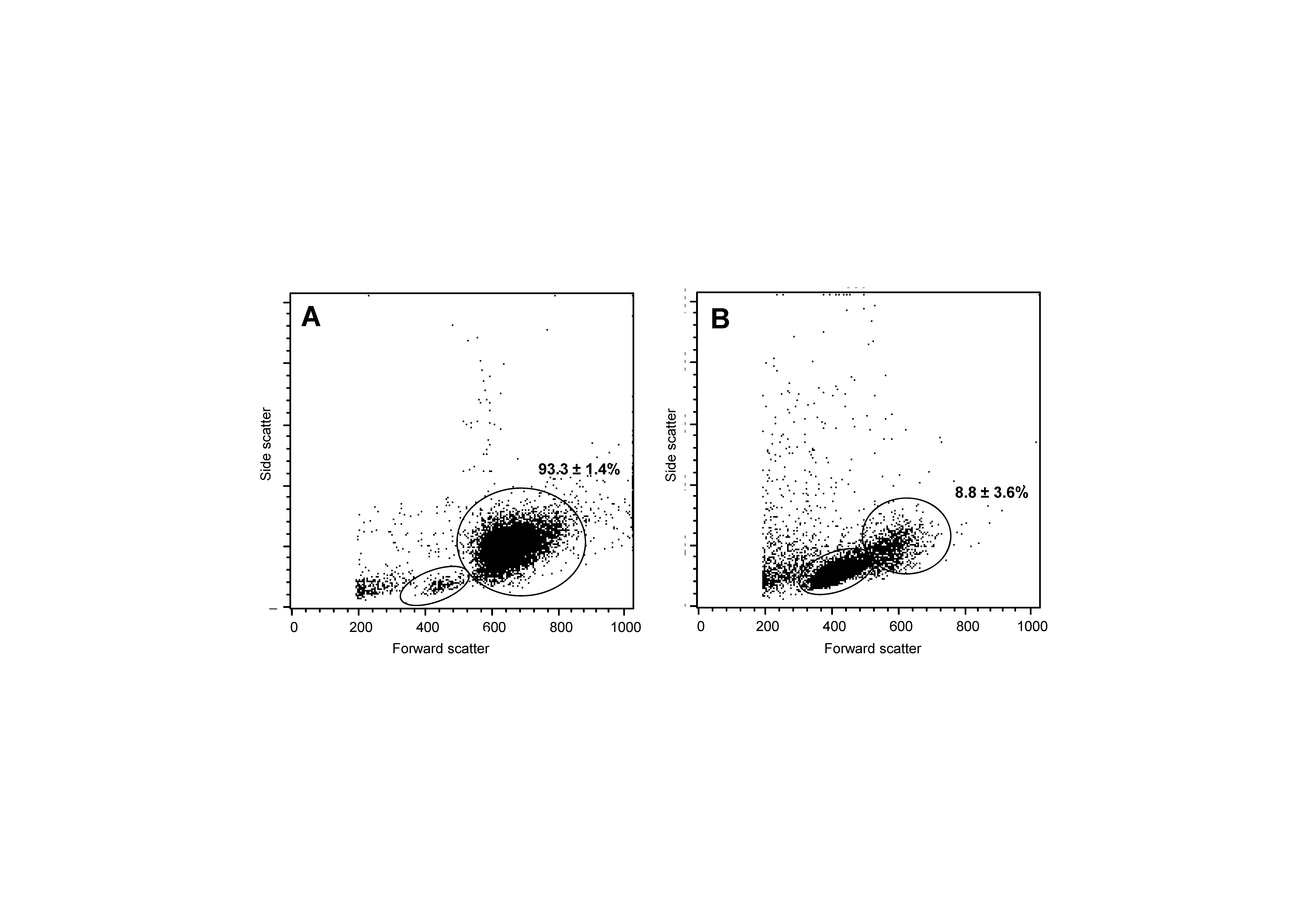

Supplement: S1 Fig — For the purification of monocytes from the peripheral blood initially a negative immuno-magnetic selection strategy was applied, offering the possibility to obtain untouched monocytes. This Monocyte Isolation Kit II (Miltenyi Biotec) is based on the magnetic labeling and depletion of non-monocytes, such as T cells, NK cells, B cells, dendritic cells and basophils, resulting in pure unlabeled monocytes. When this strategy was applied on the peripheral blood mononuclear cells of a hemodialysis patient, it resulted in a monocyte population that was 93.3 ± 1.4% pure (A). However, flow cytometric analysis revealed that the remaining lymphocytic cell fraction (depleted) still contained a monocyte fraction of 8.8 ± 3.6% monocytes (B). This was due to the fact that the Monocyte Isolation Kit II contains anti-CD16 antibodies, for depletion of granulocytes and NK cells resulting in a loss of CD16 positive monocytes, a fraction of interest in the context of inflammation, since they remain in the lymphocyte fraction. Therefore this method was not used for monocyte isolation, after which we switched to a positive selection of monocytes using CD14 MicroBeads which resulted in a isolated fraction containing CD16 positive monocytes. It is of note that, due to the monocyte heterogeneity, the choice of a correct isolation procedure, resulting in only/all the cells of interest is of great importance. The aim was to study the differential expression in the total monocyte population of hemodialysis patients compared to controls and for that purpose the use of the Monocyte Isolation Kit II, resulting only in CD16- monocytes, is not recommended. (TIFF) [file pone.0121750.s001.tiff]
