# Supplementary material for: Transcriptome Analysis in Patients with Chronic Kidney Disease on Hemodialysis Disclosing a Key Role for CD16+CX3CR1+ Monocytes
Source: PLoS One. 2015 Apr 1;10(4):e0121750. doi: 10.1371/journal.pone.0121750 (PMC4382044; doi:10.1371/journal.pone.0121750)
Supplement: S1 Table — (PDF) [file pone.0121750.s002.pdf]

Control vs CVE

Symbol	Gene name	FC	p-value	FDR
ANKFY1_2	ankyrin repeat and FYVE domain containing 1	0,50	0,030	0,644
AVPR1B	arginine vasopressin receptor 1B	1,51	0,041	0,644
CCL17	chemokine (C-C motif) ligand 17	1,72	0,007	0,644
CD209	CD209 antigen	0,55	0,027	0,644
CD244	CD244 natural killer cell receptor 2B4	0,36	0,003	0,644
CGGBP1	CGG triplet repeat binding protein 1	0,52	0,045	0,644
CMIM2	meteorin, glial cell differentiation regulator	1,50	0,041	0,644
CSF1_2	colony stimulating factor 1 (macrophage)	1,67	0,034	0,644
CXCL3	chemokine (C-X-C motif) ligand 3	1,69	0,041	0,644
DDX6	DEAD (Asp-Glu-Ala-Asp) box polypeptide 6	0,54	0,009	0,644
EDN2	endothelin 2	1,58	0,018	0,644
EGFR_4	epidermal growth factor receptor (erythroblastic leukemia viral (v-erb-b) oncogene homolog, avian)	0,59	0,018	0,644
FCGR1A	Fc fragment of IgG, high affinity Ia, receptor for (CD64)	0,46	0,034	0,644
GLG1	golgi apparatus protein 1	1,76	0,041	0,644
ICOSL	inducible T-cell co-stimulator ligand	2,43	0,027	0,644
LOC56267	hypothetical protein 669	1,59	0,009	0,644
MPO	myeloperoxidase	0,58	0,014	0,644
NCOA3	nuclear receptor coactivator 3	0,49	0,027	0,644
NR4A1_1	nuclear receptor subfamily 4, group A, member 1	0,58	0,027	0,644
NRP1	neuropilin 1	0,61	0,041	0,644
PDGFA	platelet-derived growth factor alpha polypeptide	1,65	0,041	0,644
PTGDR	prostaglandin D2 receptor (DP)	0,47	0,034	0,644
RUNX1	runt-related transcription factor 1 (acute myeloid leukemia 1; aml1 oncogene)	0,59	0,011	0,644
SDHA	succinate dehydrogenase complex, subunit A, flavoprotein (Fp)	0,62	0,009	0,644
STK4	serine/threonine kinase 4	1,68	0,041	0,644
THRAP4	thyroid hormone receptor associated protein 4	0,60	0,041	0,644
TIMP1	tissue inhibitor of metalloproteinase 1 (erythroid potentiating activity, collagenase inhibitor)	0,56	0,027	0,644

Control vs CKD5HD

Symbol	Gene name	FC	p-value	FDR
ADORA1	adenosine A1 receptor	2,00	0,045	0,827
C10orf70	chromosome 10 open reading frame 70	0,58	0,006	0,587
CASP8_E	caspase 8, apoptosis-related cysteine protease	0,51	0,001	0,535
CD244	CD244 natural killer cell receptor 2B4	0,46	0,009	0,587
CUL3	cullin 3	0,65	0,003	0,535
CX3CR1	chemokine (C-X3-C motif) receptor 1	1,78	0,011	0,667
GLA	galactosidase, alpha	2,49	0,003	0,535
IL2RG	interleukin 2 receptor, gamma (severe combined immunodeficiency)	1,94	0,018	0,667
IL8RA	interleukin 8 receptor, alpha	2,41	0,022	0,667
ITGB2	integrin, beta 2 (antigen CD18 (p95), lymphocyte function-associated antigen 1; macrophage antigen 1 (mac-1) beta subunit)	1,74	0,014	0,667
MAD	MAX dimerization protein 1	0,57	0,027	0,667
NCOA3	nuclear receptor coactivator 3	0,67	0,022	0,667
OR10H3	olfactory receptor, family 10, subfamily H, member 3	0,57	0,025	0,667
TF	transferrin	0,48	0,003	0,535
THRAP4	thyroid hormone receptor associated protein 4	0,60	0,008	0,587
TNFRSF6	tumor necrosis factor receptor superfamily, member 6	1,95	0,016	0,667
TNFRSF7	tumor necrosis factor receptor superfamily, member 7	0,55	0,037	0,827
TPST1	tyrosylprotein sulfotransferase 1	0,59	0,025	0,667

Control vs CKD5HD/CVE

Symbol	Gene name	FC	p-value	FDR
ACTB	actin, beta	1,92	0,022	0,287
ADAMDEC1	ADAM-like, decysin 1	1,65	0,018	0,287
ADORA3	adenosine A3 receptor	0,59	0,027	0,324
AKNA	AT-hook transcription factor AKNA	1,57	0,018	0,287
AKT1	v-akt murine thymoma viral oncogene homolog 1	0,41	0,009	0,287
ALOX15	arachidonate 15-lipoxygenase	0,52	0,018	0,287
AP1B1	adaptor-related protein complex 1, beta 1 subunit	1,60	0,004	0,287
CAPG	capping protein (actin filament), gelsolin-like	1,59	0,022	0,310
CCL17	chemokine (C-C motif) ligand 17	1,60	0,022	0,310
CCNL1	cyclin L1	1,53	0,022	0,310
CD244	CD244 natural killer cell receptor 2B4	0,63	0,007	0,287
CD37	CD37 antigen	1,52	0,002	0,267
CD44	CD44 antigen (homing function and Indian blood group system)	1,57	0,014	0,287
CDKN1A	cyclin-dependent kinase inhibitor 1A (p21, Cip1)	2,19	0,001	0,267
CGGBP1	CGG triplet repeat binding protein 1	0,54	0,010	0,287
CLECSF14	C-type (calcium dependent, carbohydrate-recognition domain) lectin, superfamily member 14 (macrophage-derived)	0,60	0,034	0,333
CSF1_2	colony stimulating factor 1 (macrophage)	1,76	0,018	0,287
CTSC_1	cathepsin C	1,67	0,007	0,287
CTSC_2	cathepsin C	1,54	0,027	0,324
CUL3	cullin 3	0,61	0,011	0,287
CX3CR1	chemokine (C-X3-C motif) receptor 1	1,73	0,041	0,343
CXXC1	CXXC finger 1 (PHD domain)	1,50	0,014	0,287
DKFZP564J157	DKFZp564J157 protein	1,63	0,011	0,287
EIF4E	eukaryotic translation initiation factor 4E	0,46	0,010	0,287
FCGR3A	Fc fragment of IgG, low affinity IIIa, receptor for (CD16)	1,91	0,022	0,310
FCGR3B	Fc fragment of IgG, low affinity IIIb, receptor for (CD16)	1,67	0,007	0,287
FTH1	ferritin, heavy polypeptide 1	1,51	0,027	0,324
GLA	galactosidase, alpha	2,13	0,010	0,287
GOSR2	golgi SNAP receptor complex member 2	1,74	0,045	0,354
HLA-A	major histocompatibility complex, class I, A	1,72	0,041	0,343
IL11RA	interleukin 11 receptor, alpha	2,24	0,003	0,267
IL12B	interleukin 12B (natural killer cell stimulatory factor 2, cytotoxic lymphocyte maturation factor 2, p40)	0,34	0,011	0,287
IL2RG	interleukin 2 receptor, gamma (severe combined immunodeficiency)	1,82	0,003	0,267
ITGB4	integrin, beta 4	0,46	0,041	0,343
LDHA	lactate dehydrogenase A	0,67	0,027	0,324
LTA	lymphotoxin alpha (TNF superfamily, member 1)	1,54	0,045	0,354
MAD	MAX dimerization protein 1	0,50	0,009	0,287
MMP15	matrix metalloproteinase 15 (membrane-inserted)	0,45	0,018	0,287
MMP3	matrix metalloproteinase 3 (stromelysin 1, progelatinase)	0,67	0,034	0,333
MMP9	matrix metalloproteinase 9 (gelatinase B, 92kDa gelatinase, 92kDa type IV collagenase)	0,59	0,011	0,287
MSL3L1_4	male-specific lethal 3-like 1 (Drosophila)	0,40	0,018	0,287
NRP2_1	neuropilin 2	1,54	0,045	0,354
PDGFB	platelet-derived growth factor beta polypeptide (simian sarcoma viral (v-sis) oncogene homolog)	0,62	0,002	0,267
PMP22	peripheral myelin protein 22	0,42	0,018	0,287
PPARD_1	peroxisome proliferative activated receptor, delta	0,66	0,006	0,287
PPID	peptidylprolyl isomerase D (cyclophilin D)	0,60	0,003	0,267
PPP5C	protein phosphatase 5, catalytic subunit	1,89	0,037	0,343
PSCD1	pleckstrin homology, Sec7 and coiled-coil domains 1 (cytohesin 1)	1,52	0,025	0,310

SFRP5	secreted frizzled-related protein 5	1,79	0,030	0,333
SN	sialoadhesin	0,61	0,027	0,324
SPINT2	serine protease inhibitor, Kunitz type, 2	1,54	0,041	0,343
STAT1_b	signal transducer and activator of transcription 1, 91kDa	1,95	0,009	0,287
STAT6	signal transducer and activator of transcription 6, interleukin-4 induced	1,53	0,041	0,343
TEGT	testis enhanced gene transcript (BAX inhibitor 1)	1,55	0,027	0,324
TF	transferrin	0,60	0,027	0,324
TIMP3	tissue inhibitor of metalloproteinase 3 (Sorsby fundus dystrophy, pseudoinflammatory)	1,88	0,011	0,287
TNFSF8	tumor necrosis factor (ligand) superfamily, member 8	1,58	0,045	0,354
USP18	ubiquitin specific protease 18	0,66	0,014	0,287

CVE vs CKD5HD

Symbol	Gene name	FC	p-value	FDR
ANKFY1_2	ankyrin repeat and FYVE domain containing 1	1,72	0,026	0,536
BCL6	B-cell CLL/lymphoma 6 (zinc finger protein 51)	2,32	0,023	0,536
C10orf70	chromosome 10 open reading frame 70	0,67	0,010	0,535
C11orf1	chromosome 11 open reading frame 1	0,60	0,049	0,537
CASP5	caspase 5, apoptosis-related cysteine protease	1,53	0,005	0,535
CD209	CD209 antigen	2,11	0,013	0,535
CDH1	cadherin 1, type 1, E-cadherin (epithelial)	1,82	0,019	0,536
CLECSF12_1	C-type (calcium dependent, carbohydrate-recognition domain) lectin, superfamily member 12	0,58	0,023	0,536
CX3CR1	chemokine (C-X3-C motif) receptor 1	1,58	0,049	0,537
DDX6	DEAD (Asp-Glu-Ala-Asp) box polypeptide 6	1,71	0,023	0,536
EPHA1	EphA1	1,72	0,013	0,535
EVI5	ecotropic viral integration site 5	0,52	0,028	0,537
EVL	Enah/Vasp-like	1,51	0,014	0,536
FCGR3A	Fc fragment of IgG, low affinity IIIa, receptor for (CD16)	2,14	0,023	0,536
FCGR3B	Fc fragment of IgG, low affinity IIIb, receptor for (CD16)	1,53	0,008	0,535
FGFR1_8	fibroblast growth factor receptor 1 (fms-related tyrosine kinase 2, Pfeiffer syndrome)	1,90	0,034	0,537
GADD45B	growth arrest and DNA-damage-inducible, beta	1,66	0,049	0,537
GLA	galactosidase, alpha	1,74	0,031	0,537
GLG1	golgi apparatus protein 1	0,58	0,004	0,535
HLA-A	major histocompatibility complex, class I, A	1,70	0,019	0,536
HLA-DRB5	major histocompatibility complex, class II, DR beta 5	1,77	0,010	0,535
IFNGR1	interferon gamma receptor 1	1,69	0,034	0,537
INHBA	inhibin, beta A (activin A, activin AB alpha polypeptide)	1,65	0,045	0,537
ITGB2	integrin, beta 2 (antigen CD18 (p95), lymphocyte function-associated antigen 1; macrophage antigen 1 (mac-1) beta subunit)	1,85	0,001	0,535
LOC374747	similar to coenzyme A diphosphatase	0,54	0,016	0,536
MAPK14_3	mitogen-activated protein kinase 14	0,59	0,003	0,535
NFATC1_2	nuclear factor of activated T-cells, cytoplasmic, calcineurin-dependent 1	1,93	0,017	0,536
NFKBIA	nuclear factor of kappa light polypeptide gene enhancer in B-cells inhibitor, alpha	2,13	0,003	0,535
PLA2G4A	phospholipase A2, group IVA (cytosolic, calcium-dependent)	1,56	0,034	0,537
PTGIR	prostaglandin I2 (prostacyclin) receptor (IP)	1,85	0,041	0,537
SEMA4A	hypothetical protein FLJ12287 similar to semaphorins	1,56	0,041	0,537
SGK	serum/glucocorticoid regulated kinase	1,68	0,049	0,537
STK4	serine/threonine kinase 4	0,49	0,010	0,535
TLR1	toll-like receptor 1	1,53	0,031	0,537
TNFRSF11A	tumor necrosis factor receptor superfamily, member 11a, activator of NFKB	0,66	0,041	0,537
TNFRSF5	tumor necrosis factor receptor superfamily, member 5	0,58	0,019	0,536
TNFSF6	tumor necrosis factor receptor superfamily, member 6	0,58	0,041	0,537

CVE vs CKD5HD/CVE

Symbol	Gene name	FC	p-value	FDR
ACTB	actin, beta	1,78	0,003	0,182
ADORA3	adenosine A3 receptor	0,64	0,005	0,182
AK2_A	adenylate kinase 2	1,86	0,023	0,209
AK2_B	adenylate kinase 2	1,88	0,014	0,188
AK3	adenylate kinase 3	2,11	0,011	0,188
ALOX15	arachidonate 15-lipoxygenase	0,53	0,008	0,182
BCL6	B-cell CLL/lymphoma 6 (zinc finger protein 51)	2,48	0,016	0,189
BMP2K_1	BMP2 inducible kinase	1,67	0,013	0,188
C11orf1	chromosome 11 open reading frame 1	0,57	0,019	0,200
CAPG	capping protein (actin filament), gelsolin-like	1,74	0,002	0,182
CASP5	caspase 5, apoptosis-related cysteine protease	1,68	0,004	0,182
CASP7	caspase 7, apoptosis-related cysteine protease	1,56	0,011	0,188
CCL25	chemokine (C-C motif) ligand 25	1,92	0,028	0,223
CCNL1	cyclin L1	1,71	0,005	0,182
CCR3	chemokine (C-C motif) receptor 3	2,74	0,002	0,182
CD209	CD209 antigen	1,96	0,038	0,259
CD244	CD244 natural killer cell receptor 2B4	1,73	0,019	0,200
CD4	CD4 antigen (p55)	1,88	0,007	0,182
CD44	CD44 antigen (homing function and Indian blood group system)	1,51	0,004	0,182
CDKN1A	cyclin-dependent kinase inhibitor 1A (p21, Cip1)	1,77	0,001	0,182
CLECSF14	C-type (calcium dependent, carbohydrate-recognition domain) lectin, superfamily member 14 (macrophage -derived)	0,52	0,004	0,182
CTSB	cathepsin B	2,00	0,023	0,209
CTSC_1	cathepsin C	1,68	0,028	0,223
CTSC_2	cathepsin C	1,71	0,028	0,223
CX3CR1	chemokine (C-X3-C motif) receptor 1	1,53	0,049	0,287
CYBB	cytochrome b-245, beta polypeptide (chronic granulomatous disease)	1,76	0,023	0,209
DDX6	DEAD (Asp-Glu-Ala-Asp) box polypeptide 6	1,67	0,023	0,209
DKFZP564J157	DKFZp564J157 protein	1,80	0,023	0,209
EEF1A1	eukaryotic translation elongation factor 1 alpha 1	1,61	0,034	0,249
FCGR2A	Fc fragment of IgG, low affinity IIa, receptor for (CD32)	1,64	0,023	0,209
FCGR3A	Fc fragment of IgG, low affinity IIIa, receptor for (CD16)	2,73	0,005	0,182
FCGR3B	Fc fragment of IgG, low affinity IIIb, receptor for (CD16)	1,86	0,001	0,182
FTH1	ferritin, heavy polypeptide 1	1,61	0,001	0,182
GADD45B	growth arrest and DNA-damage-inducible, beta	1,68	0,001	0,182
GOSR2	golgi SNAP receptor complex member 2	1,85	0,014	0,188
HLA-A	major histocompatibility complex, class I, A	1,94	0,003	0,182
HMGA2	high mobility group AT-hook 2	0,65	0,008	0,182
IFIX	interferon-inducible protein X	0,67	0,041	0,259
IL12B	interleukin 12B (natural killer cell stimulatory factor 2, cytotoxic lymphocyte maturation factor 2, p40)	0,33	0,019	0,200
IL6ST	interleukin 6 signal transducer (gp130, oncostatin M receptor)	2,49	0,002	0,182
INHBA	inhibin, beta A (activin A, activin AB alpha polypeptide)	1,75	0,045	0,280
IRF4	interferon regulatory factor 4	0,53	0,038	0,259
ITGB4	integrin, beta 4	0,46	0,023	0,209
IVD	isovaleryl Coenzyme A dehydrogenase	1,59	0,016	0,189
LOC374747	similar to coenzyme A diphosphatase	0,54	0,008	0,182
LTB4R	leukotriene B4 receptor	1,97	0,041	0,259
MAD	MAX dimerization protein 1	0,65	0,010	0,188
MAPK14_3	mitogen-activated protein kinase 14	0,59	0,006	0,182
MMP14	matrix metalloproteinase 14 (membrane-inserted)	0,63	0,001	0,182
MMP15	matrix metalloproteinase 15 (membrane-inserted)	0,40	0,016	0,189

MMP9	matrix metalloproteinase 9 (gelatinase B, 92kDa gelatinase, 92kDa type IV collagenase)	0,63	0,041	0,259
MSL3L1_4	male-specific lethal 3-like 1 (Drosophila)	0,42	0,028	0,223
NFATC1_2	nuclear factor of activated T-cells, cytoplasmic, calcineurin-dependent 1	1,68	0,013	0,188
NFATC2	nuclear factor of activated T-cells, cytoplasmic, calcineurin-dependent 2	0,64	0,010	0,188
NFKBIA	nuclear factor of kappa light polypeptide gene enhancer in B-cells inhibitor, alpha	1,60	0,049	0,287
NKX2-8	NK2 transcription factor related, locus 8 (Drosophila)	2,33	0,041	0,259
NRP2_1	neuropilin 2	1,59	0,028	0,223
PDCD1LG2	programmed cell death 1 ligand 2	1,52	0,028	0,223
PLA2G4A	phospholipase A2, group IVA (cytosolic, calcium-dependent	1,66	0,023	0,209
PLAT	plasminogen activator, tissue	0,65	0,019	0,200
PLEC1	plectin 1, intermediate filament binding protein 500kDa	0,64	0,028	0,223
PSAP	prosaposin (variant Gaucher disease and variant metachromatic leukodystrophy)	1,88	0,034	0,249
PSCD1	pleckstrin homology, Sec7 and coiled-coil domains 1 (cytohesin 1)	2,05	0,007	0,182
PTGES2	prostaglandin E synthase 2	1,91	0,034	0,249
PTGIR	prostaglandin I2 (prostacyclin) receptor (IP)	1,86	0,013	0,188
RAP2B	RAP2B, member of RAS oncogene family	1,64	0,041	0,259
RUNX1	runt-related transcription factor 1 (acute myeloid leukemia 1; aml1 oncogene)	1,55	0,038	0,259
S100A8	S100 calcium binding protein A8 (calgranulin A)	2,17	0,013	0,188
SEMA4A	hypothetical protein FLJ12287 similar to semaphorins	1,73	0,013	0,188
SGK	serum/glucocorticoid regulated kinase	1,66	0,023	0,209
SLAMF1	signaling lymphocytic activation molecule family member 1	1,77	0,031	0,249
SN	sialoadhesin	0,57	0,019	0,200
SRC	v-src sarcoma (Schmidt-Ruppin A-2) viral oncogene homolog (avian)	1,71	0,031	0,241
STAT1_b	signal transducer and activator of transcription 1, 91kDa	2,30	0,003	0,182
STAT6	signal transducer and activator of transcription 6, interleukin-4 induced	1,63	0,038	0,259
STK4	serine/threonine kinase 4	0,45	0,010	0,188
TBX21	T-box 21	1,79	0,014	0,189
TCEB2	transcription elongation factor B (SIII), polypeptide 2 (18kDa, elongin B)	2,23	0,010	0,188
TEGT	testis enhanced gene transcript (BAX inhibitor 1)	1,65	0,007	0,182
TIMP1	tissue inhibitor of metalloproteinase 1 (erythroid potentiating activity, collagenase inhibitor)	1,75	0,016	0,189
TIMP3	tissue inhibitor of metalloproteinase 3 (Sorsby fundus dystrophy, pseudoinflammatory)	1,88	0,008	0,182
TNFRSF11A	tumor necrosis factor receptor superfamily, member 11a, activator of NFKB	0,66	0,034	0,249
TNFRSF5	tumor necrosis factor receptor superfamily, member 5	0,54	0,049	0,287
YWHAZ	tyrosine 3-monooxygenase/tryptophan 5-monooxygenase activation protein, zeta polypeptide	1,72	0,003	0,182
ZNF9	zinc finger protein 9 (a cellular retroviral nucleic acid binding protein)	1,51	0,004	0,182

CKD5HD vs CKD5HD/CVE

Symbol	Gene name	FC	p-value	FDR
ATF3	activating transcription factor 3	1,53	0,034	1,000
ATP6V1A	ATPase, H+ transporting, lysosomal 70kDa, V1 subunit A	1,88	0,026	1,000
BMP2K_1	BMP2 inducible kinase	1,66	0,045	1,000
CDKN1A	cyclin-dependent kinase inhibitor 1A (p21, Cip1)	1,69	0,002	1,000
CTSB	cathepsin B	1,75	0,034	1,000
CTSC_1	cathepsin C	2,12	0,005	1,000
CTSC_2	cathepsin C	1,97	0,013	1,000
IL11RA	interleukin 11 receptor, alpha	1,87	0,031	1,000
LAT	linker for activation of T cells	0,51	0,021	1,000
PBX2	pre-B-cell leukemia transcription factor 2	1,61	0,023	1,000
PSCD1	pleckstrin homology, Sec7 and coiled-coil domains 1 (cytohesin 1)	1,75	0,041	1,000
SLAMF1	signaling lymphocytic activation molecule family member 1	1,59	0,045	1,000
SRC	v-src sarcoma (Schmidt-Ruppin A-2) viral oncogene homolog (avian)	1,89	0,034	1,000
TIMP3	tissue inhibitor of metalloproteinase 3 (Sorsby fundus dystrophy, pseudoinflammatory)	1,97	0,003	1,000
